# The association between cardiovascular fitness and sustained attention in the elderly—a diffusion decision model approach

**DOI:** 10.3389/fpsyg.2026.1960942

**Published:** 2026-10-01

**Authors:** Chen Chang, Chiung-Ling Chu, Yuan-Jun Deng, Tsung-Min Hung

**Affiliations:** 1 Department of Physical Education and Sports Sciences, National Taiwan Normal University, Taipei, Taiwan; 2 Institute for Research Excellence in Learning Science, National Taiwan Normal University, Taipei, Taiwan; 3 Lifestyles of Health and Sustainability Executive Master of Business Administration, National Taiwan Normal University, Taipei, Taiwan

**Keywords:** cardiovascular fitness, diffusion decision model, elderly, sustained attention, vigilance

## Abstract

**Introduction:**

Sustained attention is essential for cognitive functioning and everyday life. However, previous findings regarding the effects of aging and the benefits of cardiovascular fitness on sustained attention remain inconsistent. Moreover, the cognitive mechanisms underlying these associations remain unclear. Therefore, in addition to conventional behavioral measures, the present study applied the diffusion decision model (DDM) to characterize the cognitive processes underlying sustained attention and examine their associations with age and cardiovascular fitness in the elderly.

**Methods:**

Eighty-eight adults (Mage = 69.1, SD = 8.4) completed a modified psychomotor vigilance task and the 2-min Step Test. Sustained attention was evaluated using conventional behavioral measures (mean reaction time, Mean-RT; coefficient of variation of reaction time, CV-RT; and the slowest 10% reaction time, Slow-RT; performance decrement of reaction time, PD-RT), and DDM-derived parameters (drift rate, boundary separation, and non-decision time). Hierarchical multiple regression analyses were performed to examine the associations of age and cardiovascular fitness with behavioral and DDM-derived outcomes.

**Results:**

Increasing age was associated with longer Mean-RT and Slow-RT, as well as greater boundary separation. Cardiovascular fitness was positively associated with drift rate after controlling for age.

**Discussion:**

These findings suggest that age-related behavioral slowing in this sample was accompanied by greater response caution, whereas higher cardiovascular fitness was selectively associated with more efficient evidence accumulation. Importantly, DDM parameter revealed fitness-related differences that were not detected by conventional behavioral measures, demonstrating the added value of computational modeling for understanding exercise-cognition relationships in aging.

## 1 Introduction

Sustained attention, also referred to as vigilance, is a fundamental component of cognition (Sarter et al., 2001). It refers to an individual’s capacity to maintain readiness to detect infrequent and unpredictable signals over prolonged periods of time (Esterman and Rothlein, 2019; Sarter et al., 2001; Staub et al., 2013). Sustained attention has been proposed to rely on tonic alerting and orienting processes, requiring the continuous integration of both top-down and bottom-up processing of task-relevant stimuli (Fortenbaugh et al., 2017; Langner and Eickhoff, 2013). Impaired sustained attention has been associated with various difficulties in everyday functioning, highlighting its importance in daily life (Sarter et al., 2001; Staub et al., 2013).

Previous studies have suggested that overall attentional functioning begins to decline around the age of 40 years (Fortenbaugh et al., 2015; Lufi and Haimov, 2019). However, the effects of aging on sustained attention have remained inconsistent (Staub et al., 2013). For example, Lufi and Haimov (2019) found that although older adults exhibited slower and more variable responses, they showed better maintenance of reaction time and reaction time variability across the task. Similarly, Vallesi et al. (2021) reported that the elderly demonstrated slower reaction time but higher accuracy during non-target trials compared to younger adults. Moreover, the elderly appeared to be less susceptible to vigilance decrement over time (Robison et al., 2022).

One possible explanation for these inconsistent findings is that conventional behavioral measures are susceptible to speed–accuracy tradeoffs. After controlling for general slowing and accuracy differences, only the alerting network exhibited a stable age-related decline (Veríssimo et al., 2022). These findings suggest the alerting network, a key process supporting sustained attention, may be particularly sensitive to age-related decline. Integrating reaction time and accuracy measures simultaneously is recommended to better characterize the underlying cognitive mechanisms (Draheim et al., 2019; Draheim et al., 2022; Heitz, 2014).

The diffusion decision model (DDM) is a computational modeling approach that jointly models distributional reaction time and response accuracy in binary decision-making task (Ratcliff and McKoon, 2008; Ratcliff and Rouder, 1998; Ratcliff et al., 2016). Moreover, this model helps further infer the latent cognitive mechanisms underlying cognitive performance (Matzke and Wagenmakers, 2009; Rieger and Miller, 2020). Specifically, the model decomposes task performance into several latent cognitive components, including evidence accumulation efficiency (drift rate, *v*), response cautiousness (boundary separation, *a*), and non-decision processes (non-decision time, *T_er_*).

Research applying the diffusion decision model (DDM) suggests that age-related slowing in processing speed may reflect changes in multiple latent cognitive mechanisms. Compared with younger adults, older adults typically demonstrate lower drift rates, greater boundary separation, and longer non-decision times, indicating reduced evidence accumulation efficiency, more cautious response strategies, and slower peripheral processing (Theisen et al., 2021; von Krause et al., 2022). Notably, declines in drift rate become more apparent after the age of 60 years, alongside substantial increases in overall reaction time (von Krause et al., 2022). More recent evidence further suggest age-related slowing may occur across both decisional and non-decisional processing components (Kelber et al., 2025). These findings highlight the value of computational approach such as the DDM for disentangling the latent cognitive mechanisms underlying age-related differences in sustained attention.

In addition to age-related differences, individual factors may contribute to variability in sustained attention performance. Cardiovascular fitness represents a potentially modifiable factor that has been associated with attentional functioning (Fernandes et al., 2019). Higher-fit individuals have consistently demonstrated faster reaction times during sustained attention tasks across different age groups, including children (Pontifex et al., 2011), adolescents (Pontifex et al., 2012; Reigal et al., 2020; Voss et al., 2011), and young adults (Ciria et al., 2017; Di Muccio et al., 2022; Di Muccio et al., 2023; Luque-Casado et al., 2020; Luque-Casado et al., 2016a, 2016b). Beyond behavioral performance, neurophysiological evidence indicates that higher-fit individuals exhibit greater attentional resource allocation during stimulus processing (Di Muccio et al., 2022; Luque-Casado et al., 2016b), and more stable attentional engagement throughout sustained attention tasks (Luque-Casado et al., 2020; Luque-Casado et al., 2016b). Furthermore, enhanced endogenous attentional preparation and increased activation of attentional networks has also been observed before stimulus onset (Di Muccio et al., 2023; Luque-Casado et al., 2016b). Consistent with these findings, higher-fit individuals exhibit greater transient cardiac deceleration following cue presentation, which has been linked to enhanced attentional preparatory states during sustained attention tasks (Luque-Casado et al., 2016a).

Despite these findings, relatively few studies have specifically examined the role of cardiovascular fitness in maintaining sustained attention among the elderly (Fernandes et al., 2019; Hajar et al., 2019) and the available findings remain inconclusive. Bunce (2001) suggested that better health-related physical fitness may attenuate age-related decline in sustained attention. However, more recent research indicated no significant association between cardiovascular fitness and sustained attention (Gongora-Meza and Sanchez-Lopez, 2024). Moreover, previous studies have primarily relied on conventional measures, making it difficult to determine which latent cognitive processes underlying sustained attention are influenced by cardiovascular fitness. Therefore, further investigation is needed to clarify the relationship between cardiovascular fitness and sustained attention in the elderly.

Accordingly, the present study applied the DDM framework to investigate latent cognitive processes underlying sustained attention performance in the elderly. Furthermore, we examined whether cardiovascular fitness was associated with DDM-derived cognitive parameters, thereby providing insight into the specific cognitive processes through which cardiovascular fitness may contribute to sustained attention performance.

## 2 Materials and methods

### 2.1 Participants

Ninety-three participants were initially recruited for the study. Participants were eligible for inclusion if they: (1) were aged 50 years or older; (2) had no history of neurodegenerative diseases; (3) had normal or corrected-to-normal vision; (4) were deemed eligible to participate in physical activity based on the Physical Activity Readiness Questionnaire (PAR-Q); and (5) were not currently taking medications known to affect the central nervous system or neurological functioning. Three participants were excluded due to inability to complete the tasks, and two were excluded due to equipment malfunction during the cognitive task. The final analysis therefore included 88 participants.

### 2.2 Measurements

#### 2.2.1 Psychomotor vigilance task

A modified version of the psychomotor vigilance task (PVT), originally developed by Dinges and Powell (1985), was applied to assess sustained attention performance. In the present study, the traditional single-stimulus PVT paradigm was adapted into a two-choice discrimination task to enable further examination of latent decision-making processes using diffusion decision modeling (DDM). Previous research has shown that choice-response is also sensitive to detecting changes in vigilance attention (Cunningham et al., 2018).

Each trial began with a fixation cross presented at the center of the screen, followed by a variable interstimulus interval (ISI) ranging from 2 to 5 s. Subsequently, either a red or blue circle appeared at the center of the display. Participants were instructed to press the left response key when a red circle appeared and the right response key when a blue circle appeared. Participants were required to respond as quickly and accurately as possible within a 1 s response window. Responses made before stimulus onset or failures to respond within the response window were classified as incorrect responses.

The task consisted of five blocks of 45 trials each, with a 30 s rest interval between blocks. The entire PVT lasted approximately 30 min.

To quantify the performance of the psychomotor vigilance task, the following measures were calculated. Mean reaction time (Mean-RT) was calculated as the mean across correct trials within the predefined response window. Response variability was assessed using the coefficient of variation of reaction time (CV-RT), calculated as the standard deviation of reaction divided by the mean reaction time and multiplied by 100. Slow-RT was calculated as the mean reaction time of the slowest 10% of valid trials for each participant and was used as an indicator of the attentional lapses (Basner and Dinges, 2011). To examine changes in performance overtime, performance decrement of reaction time (PD-RT) was calculated based on the change in mean reaction time from the first to the final block using the formula [(Block 5-Block1)/Block1] × 100 (Di Muccio et al., 2022), with positive PD-RT values indicating greater slowing in reaction time from the beginning to the end of the task.

#### 2.2.2 Physical fitness test

The physical fitness test was measured through the Senior Physical Fitness Test (Rikli and Jones, 2013). Specifically, the entire fitness test is consisted of 7 subtests for four main physical outcomes, including muscle endurance, flexibility, agility and balance, and the cardiovascular ability.

#### 2.2.3 Muscle endurance

Arm curls and chair sit-to-stand are measured as the muscle endurance ability for the upper and lower limb.

For the arm curls, the participants are instructed to sit on a chair while performing arm curl using their dominant hand with a dumbbell (5 pounds for females, 8 pounds for males). The arm must be fully bent and straightened at the elbow, and the upper arm is braced and closed to the body so that only the lower arm can move. The repetitions of the movement in 30 s are recorded.

As for the chair sit-to-stand, the participants are instructed to sit in the middle of a chair with their arm crossed on the opposite shoulders. Keeping their feet flat on the floor with a straight back, they repeat the full standing position and sit back down for 30 s. The repetitions of the movement in 30 s are recorded.

##### 2.2.3.1 Flexibility

Back scratch and the chair sit-and-reach are measured as the flexibility ability for the upper and lower limb.

For the back scratch, the participants remained in a standing position and were instructed to place one hand behind their head and the other behind their back, reaching as far as possible to touch the palm or overlap the middle fingers of both hands. The best score to the nearest centimeter is recorded.

As for the chair sit-and-reach test, the participants remained sit on the edge of a chair while one leg extended forward with straight knee and the ankle bent at 90 degrees, and the other remains flat on the floor. The participants are instructed to cross both hands and even their middle fingers. Then, they reach forward as far as possible toward their toes. If the participants cannot reach their toes, the distance between the fingertips and toes is recoded as a negative score. In contrast, if the participant’s fingertips overlap with their toes, their overlapping distance is recorded positively.

##### 2.2.3.2 Agility and balance

The stationary balance ability is assessed through balance on one leg with eyes open. The participants are instructed to stand on one leg with their eyes opened. The duration for which the participants perform standing with one-leg without hopping or putting their raised foot down is recorded in seconds.

The dynamic balance and agility are measured through 8-foot up-and-go. A cone is placed 8 feet in front of a chair. Participants fully sit on the chair with their hands resting on their knees and their feet flat on the floor. The time from sitting, to moving around the cone, returning to the chair, and then back to the starting position is recorded in second.

##### 2.2.3.3 Cardiovascular ability

A 2-min Step is measured to assess the cardiovascular capacity. A customed height of the step standard is measure by the half point from iliac crest to the patella. The number of times the right knee reaches the required height for 2 min is recorded.

#### 2.2.4 DDM parameter modeling

Reaction time data were analyzed using the Wiener diffusion decision model (DDM) implemented within a Bayesian framework using the brms package in R (Bürkner, 2017). Prior to modeling, trials with missing responses or reaction times, reaction times shorter than 200 ms, and responses other than the predefined response keys were excluded. Reaction times were converted from milliseconds to seconds before model fitting.

Due to the high response accuracy observed in the task (95.5%), decision boundaries were specified according to response type (i.e., upper vs. lower response boundary corresponding to the two response keys). This approach allowed the diffusion process to be estimated using the two response alternatives while minimizing instability associated with sparse error trials.

Individual-level DDMs were estimated separately for each participant using the Wiener likelihood function (Myers et al., 2022). The model estimated four parameters: drift rate (*v*), boundary separation (*a*), non-decision time (*T_er_*), and starting-point bias (*z*) (Ratcliff and McKoon, 2008). Weakly informative priors were specified for all parameters based on plausible ranges commonly adopted in diffusion modeling studies. Specifically, a normal prior was assigned to the intercept (drift rate), a gamma prior to boundary separation, a normal prior to non-decision time, and a beta prior to response bias.

Bayesian estimation was conducted using Markov chain Monte Carlo sampling with four chains, 4,000 iterations per chain, and 2,000 warm-up iterations. Model convergence was evaluated using the potential scale reduction factor (R̂), effective sample size (ESS), and posterior predictive checks (Gelman et al., 2014; Vehtari et al., 2021). Convergence was considered acceptable when R̂ values approached 1.00 and effective sample sizes were sufficiently large (see Supplementary Table 1).

Four diffusion model parameters (drift rate, boundary separation, non-decision time, and starting point bias) were estimated. Three parameters (drift rate, boundary separation, and non-decision time) were further analyzed as these parameters are primarily used in the context of aging research (Ratcliff et al., 2010).

#### 2.2.5 Procedure

After providing informed consent, participants first completed a demographic questionnaire, including information regarding age, sex, and years of education. Participants also completed the Physical Activity Readiness Questionnaire (PAR-Q) and International Physical Activity Questionnaire (IPAQ) to assess eligibility to participate in exercise and their habitual physical activity levels.

Subsequently, participants performed the modified psychomotor vigilance task (PVT) to assess sustained attention performance. Following completion of the cognitive assessment, participants underwent a series of physical fitness assessments. Short rest periods were provided between assessments when necessary to minimize excessive fatigue. The entire experimental procedure lasted approximately 90 min.

#### 2.2.6 Statistical analysis

All statistical analyses were conducted using JASP (Version 0.95.1) and R software (2026.04.0 + 526).

Descriptive statistics were first calculated for all study variables. To further examine whether physical fitness measures predicted DDM parameters beyond the effects of age, hierarchical multiple regression analyses were performed. Age was entered in Step 1 as covariates, followed by physical fitness variables in Step 2. Separate regression models were conducted for each DDM parameter.

Model assumptions were evaluated prior to the interpretation. Normality of residuals and homoscedasticity were assessed by the visual inspection of normal Q–Q plot and residuals versus fitted plots, respectively (see Supplementary Figures 1, 2). Multicollinearity was evaluated using variance inflation factors (VIFs), and the independence of residuals was assessed using the Durbin–Watson statistic (see Supplementary Table 2). Statistical significance was set at *p* < 0.05.

## 3 Results

Figure 1 presents the flow diagram, and Table 1 summarizes the demographic characteristics of the participants.

**Figure 1:**
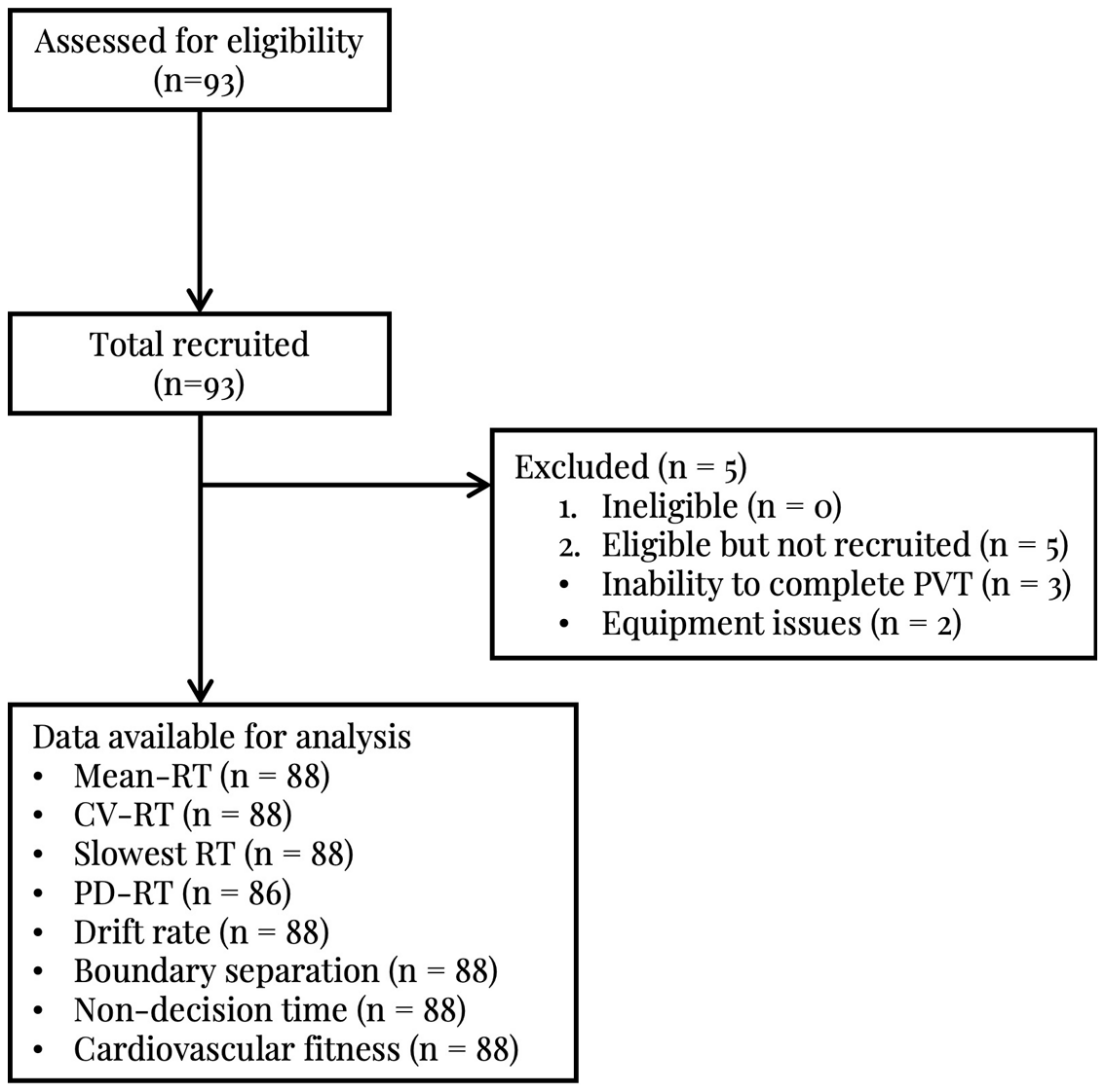
Participant flow diagram. 2-Min, 2-min Step Test; Mean-RT, mean reaction time; CV-RT, coefficient of variation of reaction time; PD-RT, performance decrement of reaction time.

**Table 1:** Participant characteristics.

Variable	Mean ± SD/*n* (%)
Age	69.1 ± 8.4
Female	63 (71.5)
Educational attainment (years)	14.2 ± 3.2
IPAQ (METs)	2348.1 ± 3018.3

IPAQ, international physical activity questionnaire.

Descriptive statistics (means and standard deviations) for age, 2-min Step Test performance, behavioral measures derived from the Psychomotor Vigilance Task (PVT), and DDM parameters are presented in Table 2.

**Table 2:** Descriptive statistics and bivariate correlations among study variables.

Variable	M	SD	Age	2-Min	Mean-RT	CV-RT	Slow-RT	PD-RT	*v*	*a*	*T_er_*
Age	69.1	8.4	-								
2-Min	99.6	17.9	−0.13	-							
Mean-RT	589.6	83.8	0.29	−0.10	-						
CV-RT	23.8	6.2	−0.16	−0.17	−0.23	-					
Slow-RT	782.7	99.2	0.26^*^	−0.15	0.94^***^	0.02	-				
PD-RT	1.9	7.8	−0.20	0.04	−0.10	0.08	−0.10	-			
*v*	1.1	0.3	0.15	0.24^*^	−0.12	−0.53^***^	−0.28^**^	−0.05	-		
*a*	1.1	0.2	0.28^**^	−0.15	0.73^***^	0.08	0.75^***^	−0.11	−0.21	-	
*T_er_*	0.4	0.1	0.12	0.06	0.58^***^	−0.50^***^	0.45^***^	−0.00	0.11	−0.12	-

M, mean; SD, standard deviation; 2-Min, 2-min Step Test; Mean-RT, mean reaction time; CV-RT, coefficient of variation of reaction time; Slow-RT, slowest 10% reaction time; PD-RT, performance decrement of reaction time; v, drift rate; a, boundary separation; T_er_, non-decision time. ^*^p < 0.05; ^**^p < 0.01; ^***^p < 0.001.

For the first research question, the baseline model (Step 1), which included age as the predictor, was statistically significant for Mean-RT, *F* (1, 86) = 8.05, *p* = 0.006, Slow-RT, *F* (1, 86) = 6.06, *p* = 0.016, and boundary separation, *F* (1, 86) = 7.19, *p* = 0.009, but not for CV-RT, PD-RT, drift rate, or non-decision time (all *ps* > 0.05). Specifically, age was a significant positive predictor of Mean-RT (β = 0.29, *p* = 0.006), Slow-RT (β = 0.26, *p* = 0.016), and boundary separation (β = 0.28, *p* = 0.009), indicating that increasing age was associated with slower reaction time and greater response caution (Figure 2).

**Figure 2:**
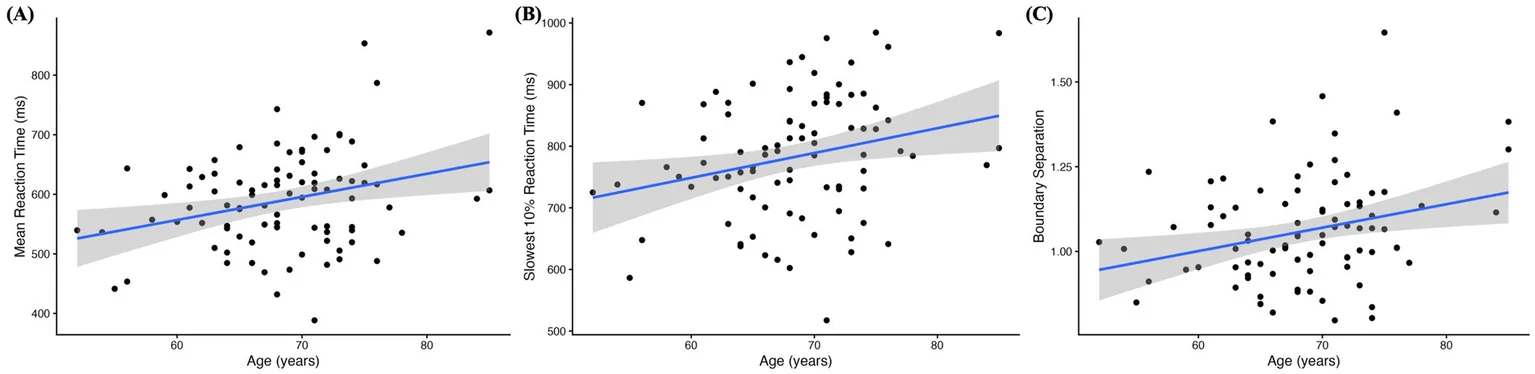
Relationships between age and sustained attention performance. Panel **(A)** Relationship between mean reaction time and age. Panel **(B)** Relationship between the slowest 10% reaction time and age. Panel **(C)** Relationship between boundary separation and age. The blue line indicates the fitted linear regression line, and the shaded region represents the 95% confidence interval.

For the second research question, the addition of 2-min Step performance explained a significant proportion of additional variance in drift rate beyond age (Δ*R*^2^ = 0.068, Δ*F* (1, 85) = 6.35, *p* = 0.014). Specifically, 2-min Step performance was a significant positive predictor of drift rate (β = 0.26, *p* = 0.014) (Figure 3), but not of Mean-RT, CV-RT, Slow-RT, PD-RT, boundary separation, or non-decision time (all *ps* > 0.05). These findings suggest that higher cardiovascular fitness was associated with more efficient evidence accumulation in the elderly.

**Figure 3:**
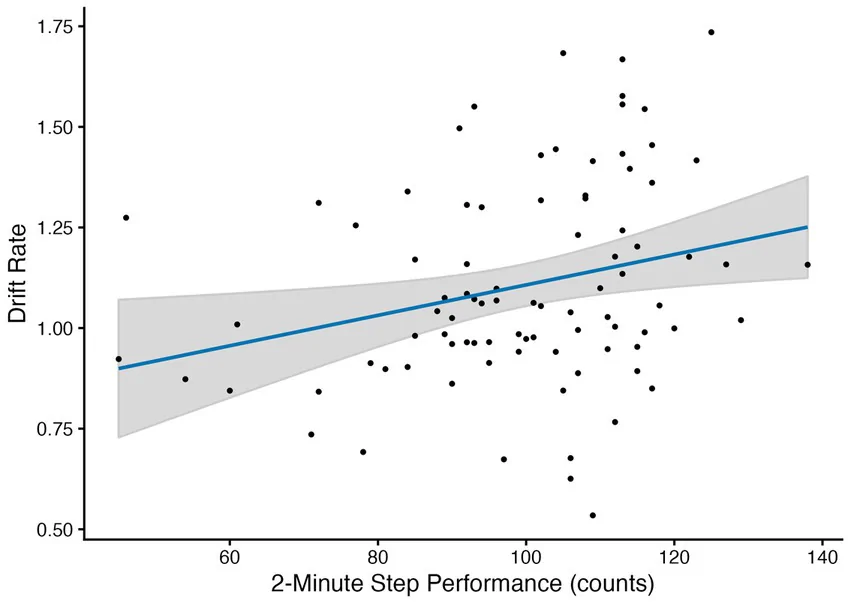
Relationships between cardiovascular fitness and drift rate. The blue line indicates the fitted linear regression line, and the shaded region represents the 95% confidence interval.

The complete results of the hierarchical regression analyses are provided in Supplementary Table 3.

## 4 Discussion

The present study examined age-related differences in sustained attention using both conventional behavioral measures and diffusion decision modeling (DDM). Overall, increasing age was associated with slower reaction time, and a more conservative response strategy, as reflected by larger boundary separation. In contrast, age was not significantly associated with response variability, performance decrement, drift rate, or non-decision time. Collectively, these findings suggest that age-related behavioral slowing within the present sample was accompanied primarily by greater response caution, whereas no significant age-related differences were detected in evidence accumulation efficiency or non-decisional processing.

At the behavioral level, the present study found that increasing age was associated with both longer mean reaction time and longer reaction times in the slowest 10% of trials. These findings are consistent with previous studies demonstrating age-related slowing during sustained attention tasks (Fortenbaugh et al., 2015; Lufi and Haimov, 2019), with age-related differences being particularly pronounced in the right tail of the reaction time distribution (Robison et al., 2022). More broadly, age-related slowing has been widely documented across multiple cognitive domains (Finkel et al., 2007; Salthouse, 1996, 2000, 2019). Together with the positive association between boundary separation and age, these findings suggest that behavioral slowing associated with increasing age may be partly attributable to a more cautious response strategy. This interpretation is consistent with previous studies reporting greater response caution among older adults (Fortenbaugh et al., 2015; Lufi and Haimov, 2019; Vallesi et al., 2021).

Contrary to our expectations, no significant association were observed between age and either CV-RT or PD-RT. Previous studies have suggested that intra-individual variability is particularly sensitive to age-related cognitive changes (MacDonald et al., 2006). However, age differences in response variability may be more evident in sustained attention paradigms that place greater demands on inhibitory control (Fortenbaugh et al., 2015; Robison et al., 2022; Yamashita et al., 2021). In addition, the elderly may adopt slower and more cautious response strategies to maintain accuracy, thereby reducing trial-to-trial fluctuations (Lufi and Haimov, 2019). Regarding the performance decrement, previous research found that older adults did not exhibit the vigilance decrement observed in younger adults, possibly because they remained more motivated throughout the task (Robison et al., 2022). However, motivation was not assessed in the present study, precluding us from determining whether motivational factors contributed to the absence of age-related performance decrement.

Similarly, no significant association between age and drift rate was observed. Although this finding differs from lifespan studies (von Krause et al., 2022; Kelber et al., 2025), it is consistent with previous DDM studies showing that age effects on drift rate are highly task dependent (Ratcliff et al., 2006, 2010; Theisen et al., 2021). Age-related differences may be attenuated in the task involving highly discriminable and sufficient sensory information (Ratcliff et al., 2001; Thapar et al., 2003). Consequently, the present task may have been insufficiently sensitive to detect age-related differences in evidence accumulation efficiency.

Another possible explanation for the absence of age-related associations in drift rate and non-decision time is the restricted age range of the present sample. Unlike lifespan studies examining age-related trajectories in diffusion model parameters (von Krause et al., 2022; Kelber et al., 2025), and studies comparing these examining the parameters across multiple age groups (Ratcliff et al., 2006, 2010; Theisen et al., 2021), the current study focused exclusively on healthy older adults. Age-related differences in diffusion model parameters were less consistent when comparisons were restricted to older and old-old adults (Ratcliff et al., 2006, 2010). Furthermore, each parameter follow distinct non-linear trajectories across development and aging (von Krause et al., 2022), suggesting that age-related variability may not be adequately captured by linear associations within a relatively restricted age range. Therefore, age-related variability in evidence accumulation and non-decision processes may have been less apparent in the present sample.

Cardiovascular fitness was positively associated with drift rate, whereas no significant associations were observed for behavioral measures of sustained attention. Within the DDM framework, drift rate reflects the efficiency with which evidence is extracted and accumulated toward a decision (Weigard et al., 2026). Therefore, the present findings suggest that cardiovascular fitness may primarily benefit the latent cognitive processes rather than observable behavioral performance.

Although the precise neural mechanisms remain unclear, previous studies have demonstrated that individuals with higher cardiovascular fitness exhibit a greater attentional allocation (Luque-Casado et al., 2016a) and a more efficient processing of task-relevant information (Di Muccio et al., 2022), as reflected by the neural activity during P3b time-window. Importantly, accumulating evidence suggests that the P3b component reflects the build-up of a decision variable during perceptual decision making, with steeper accumulation trajectories associated with faster decision formation (Twomey et al., 2015). More recently, the centroparietal positivity (CPP) has been identified as a neural signature closely associated with evidence accumulation, with its build-up rate showing strong correspondence to computational measures of evidence accumulation (Kelly and O’Connell, 2015; O'Connell et al., 2012). Consequently, the positive association between cardiovascular fitness and drift rate observed in the present study may indicate that higher levels of fitness facilitate evidence accumulation. Future studies combining electrophysiological measures with diffusion decision modeling are needed to verify this interpretation.

The absence of significant associations between cardiovascular fitness and behavioral measures of sustained attention may suggest that behavioral performance is influenced by factors beyond aerobic fitness alone. One possible explanation is that the cognitive demands embedded within habitual physical activity may play an important role in shaping attentional performance. Wang and Guo (2020) found that older adults participating in open-skill sports demonstrated superior executive attention compared with both closed-skill athletes and sedentary individuals, whereas no differences were observed between the latter two groups. Similar findings have also been reported in children (Ballester et al., 2018), adolescents (Ballester et al., 2015), and young adults (Ballester et al., 2019; Pérez et al., 2014; Sanabria et al., 2019). Notably, Ballester et al. (2019) found that hand-eye coordination was a stronger predictor of sustained attention performance than cardiovascular fitness. Together with the present findings, these studies suggest that while cardiovascular fitness may contribute to the efficiency of evidence accumulation, behavioral performance during sustained attention may additionally depend on the cognitive demands imposed by long-term exercise experience. Similarly, the absence of an association between cardiovascular fitness and PD-RT is consistent with previous studies suggesting that higher fitness was not associated with better maintenance of behavioral performance over time in young adults (Di Muccio et al., 2022; Luque-Casado et al., 2016b). However, evidence in the elderly suggests that fitness-related benefits may be more apparent under more demanding tasks (Bunce, 2001). Therefore, whether cardiovascular fitness contribute to the maintenance of sustained attention warrants further investigation.

Cardiovascular fitness was not significantly associated with boundary separation, suggesting that fitness-related differences in sustained attention were unlikely to be explained by variations in response caution or decision strategy. Although direct evidence linking cardiovascular fitness to boundary separation remains limited, Bunce (2001) similarly reported no association between cardiovascular fitness and response bias during a vigilance task. While response bias and boundary separation are conceptually distinct, both reflect strategic aspects of response behavior. Together, these findings provide little support for the notion that cardiovascular fitness influences sustained attention through changes in response caution or strategic response settings.

Cardiovascular fitness was not significantly associated with non-decision time. Interpretation of this null finding should be made cautiously because non-decision time is a composite parameter that encompasses perceptual encoding and motor execution, processes that may be differentially influenced by cardiovascular fitness. Previous studies have reported that higher cardiovascular fitness is associated with enhanced neural motor preparation (Fröhlich et al., 2024; Kamijo et al., 2010; Luque-Casado et al., 2016a), whereas evidence supports beneficial effects on stimulus encoding (Fröhlich et al., 2024; Wu et al., 2022) or motor execution (Brush et al., 2020) were less consistent. Consequently, the absence of an association between cardiovascular fitness and non-decision time may reflect the heterogeneous nature of this parameter rather than the absence of fitness-related effects on all non-decisional processes. Future studies incorporating neurophysiological measures may help dissociate these component processes and clarify the specific non-decisional stages through which cardiovascular fitness influences cognitive performance.

Beyond the neurophysiological evidence discussed above, the locus coeruleus–norepinephrine (LC–NE) system may provide a broader theoretical framework for understanding the relationship between cardiovascular fitness and sustained attention performance. The LC–NE system plays a critical role in sustaining attention by regulating alertness, attentional stability, and task engagement (Esterman and Rothlein, 2019; Fortenbaugh et al., 2017; Torres et al., 2025). Importantly, the locus coeruleus is considered one of the brain regions most vulnerable to age-related degeneration (Mather and Harley, 2016), and greater LC integrity has been associated with better attentional performance in the elderly (Plini et al., 2021). Consistent with this possibility, Plini et al. (2024) reported that higher cardiovascular fitness was associated with greater LC integrity in the elderly. Greater LC integrity may support more efficient noradrenergic modulation, leading to a higher signal-to-noise ratio of task-relevant information and, consequently, more sustained attention performance (Robertson, 2013). Taken together, these findings suggest that the LC–NE system may represent one potential mechanism linking cardiovascular fitness to sustained attention. Future studies integrating structural and functional neuroimaging together with neurophysiological measures are needed to further establish the relationships among cardiovascular fitness, LC integrity, neural activity, and latent decision-making processes.

Although the present study provides novel insights into the relationships among aging, cardiovascular fitness, and sustained attention, several limitations should be acknowledged. First, the cross-sectional design precludes causal inference regarding the relationship between cardiovascular fitness and latent cognitive processes. Future longitudinal and intervention-based studies are needed to determine whether improvements in cardiovascular fitness led to enhanced evidence accumulation efficiency in the elderly. Second, sustained attention performance is influenced by multiple psychological states, including arousal, motivation, and mind wandering (Esterman and Rothlein, 2019). Although these factors were not directly assessed in the present study, they may influence both behavioral performance and computational measures. Future studies incorporating these variables may provide a more comprehensive understanding of the relationships among aging, cardiovascular fitness, and sustained attention. Furthermore, the response window of the present study was limited to 1,000 ms. Consequently, responses beyond this time window were not recorded, preventing the present study from fully examining the right tail of the reaction time distribution. Third, the present study focused on decomposing sustained attention performance using diffusion decision modeling. Although this approach provides valuable insight into latent decision-making processes, it does not fully capture the temporal dynamics of sustained attention. For example, variance time course analyses quantify moment-to-moment fluctuations in attentional stability and may provide complementary information regarding attentional dynamics (Esterman and Rothlein, 2019). Integrating multiple analytical approaches may therefore provide a more comprehensive characterization of sustained attention. Fourth, the 2-min step test is a practical and widely used field test for estimating cardiovascular fitness in healthy adults (Bennett et al., 2016; Surapichpong et al., 2024); and older adults (Berlanga et al., 2023). However, its validity and responsiveness require further evaluation (Crouch and Bohannon, 2019). Future studies may benefit from incorporating direct assessments of maximal oxygen uptake (VO₂max) to provide a more precise estimate of cardiovascular fitness. Lastly, the present study did not collect information regarding participants’ previous sports participation and exercise experience. Previous research has indicated that different types of exercise may have differential effect on various aspects of cognitive function in the elderly (Ingold et al., 2020). Specifically, open-skilled exercise may confer greater cognitive benefits than closed-skill exercise potentially due to the additional requirements of continuously adapting to changing environments and greater demands on information-processing speed (Gu et al., 2019). However, the mechanisms underlying these differential effects warrant further investigation.

In conclusion, the present study demonstrated that cardiovascular fitness was selectively associated with drift rate, but not with boundary separation, non-decision time, or starting point bias, in the elderly performing a sustained attention task. These findings suggest that better cardiovascular fitness may specifically support attentional evidence accumulation efficiency rather than generalized motor or strategic aspects of decision-making. The application of diffusion decision modeling further highlights the importance of decomposing behavioral performance into latent cognitive components when investigating exercise–cognition relationships in aging populations.

## Data Availability

The original contributions presented in the study are included in the article/Supplementary material, further inquiries can be directed to the corresponding author.
