# Supplementary material for: The association between cardiovascular fitness and sustained attention in the elderly—a diffusion decision model approach
Source: Front Psychol. 2026 Oct 1;17:1960942. doi: 10.3389/fpsyg.2026.1960942 (PMC13668527; doi:10.3389/fpsyg.2026.1960942)
Supplement: Supplementary file 1 [file Supplementary_file_1.DOCX]

**Supplementary Table 1.**

*Bayesian Convergence Diagnostics for Diffusion Decision Model Parameters*

| Parameter | R-hat (Median [Min, Max]) | Bulk ESS (Median [Min, Max]) | Tail ESS (Median [Min, Max]) |
| --- | --- | --- | --- |
| Drift rate (*v*) | 1.001 [1.000, 1.002] | 4,492.6 [3633.9, 5669.9] | 5.172.0 [4.362.4, 5790.9] |
| Boundary separation (*a*) | 1.000 [1.000, 1.001] | 5503.1 [3994.7, 6634.9] | 5230.3 [4388.7, 5930.5] |
| Non-decision time (*T_er_*) | 1.000 [1.000, 1.002] | 5133.4 [3505.3, 6301.9] | 5252.2 [4393.7, 6009.5] |
| Starting point (*z*) | 1.001 [1.000, 1.002] | 4392.5 [3557.2, 5657.3] | 5045.2 [3964.1, 6004.6] |

Note. Values are presented as median [minimum, maximum]. R-hat = potential scale reduction factor; Bulk ESS = bulk effective sample size; Tail ESS = tail effective sample size. R-hat values close to 1.00 and large ESS values indicate satisfactory convergence and efficient posterior sampling.

**Supplementary Figure 1.**

*Normal Q-Q Plots of Standardized Residuals for the Final Regression Models*

*Note.* Panels A–G correspond to the models for mean reaction time, coefficient of variation of reaction time, slowest 10% reaction time, performance decrement of reaction time, drift rate, boundary separation, and non-decision time, respectively.

**Supplementary Figure 2.**

*Residuals versus Fitted Values for the Final Hierarchical Regression Models*

*Note.* Panels A–G correspond to the models for mean reaction time, coefficient of variation of reaction time, slowest 10% reaction time, performance decrement of reaction time, drift rate, boundary separation, and non-decision time, respectively.

**Supplementary Table 2.**

| Outcome | Durbin–Watson | *p* |
| --- | --- | --- |
| Mean-RT | 1.69 | .130 |
| CV-RT | 1.96 | .829 |
| Slow-RT | 1.88 | .534 |
| PD-RT | 2.31 | .156 |
| Drift rate (*v*) | 2.16 | .470 |
| Boundary separation (*a*) | 2.13 | .547 |
| Non-decision time (*T_er_*) | 1.84 | .423 |

*Regression Diagnostic Statistics for the Final Hierarchical Regression Models*

*Note.* Mean-RT = mean reaction time; CV-RT = coefficient of variation of reaction time; Slow-RT = slowest 10% reaction time; PD-RT = performance decrement of reaction time. All final models included age and 2-minute step performance as predictors. Variance inflation factors (VIFs) ranged from 1.01 to 1.02, with tolerance values ranging from .984 to .987.

**Supplementary Table 3.**

*Hierarchical Regression Analyses Examining the Associations of Age and Cardiovascular Fitness with Behavioral and Diffusion Model Outcomes*

| Outcome | Step | Predictor | B | β | 95% CI for B | *p* | *R*² | Δ*R*² | Δ*F* |
| --- | --- | --- | --- | --- | --- | --- | --- | --- | --- |
| Behavioral measures | | | | | | | | | |
| Mean-RT | 1 | Age | 3.88 | 0.29 | [1.16, 6.60] | 0.006 | 0.086 | — | 8.05** |
|  | 2 | Age | 3.78 | 0.29 | [1.03, 6.53] | 0.008 | 0.089 | 0.003 | 0.32 |
|  |  | 2-Min | −0.28 | −.06 | [−1.25, 0.70] | 0.572 |  |  |  |
| CV-RT | 1 | Age | −0.16 | −.16 | [−0.36, 0.05] | 0.145 | 0.025 | — | 2.17 |
|  | 2 | Age | −0.18 | −.18 | [−0.39, 0.03] | 0.091 | 0.061 | 0.036 | 3.26 |
|  |  | 2-Min | −0.07 | −.19 | [−0.14, 0.01] | 0.074 |  |  |  |
| Slow-RT | 1 | Age | 4.03 | 0.26 | [0.77, 7.28] | 0.016 | 0.066 | — | 6.06* |
|  | 2 | Age | 3.79 | 0.24 | [0.52, 7.07] | 0.024 | 0.079 | 0.013 | 1.24 |
|  |  | 2-Min | −0.65 | −.12 | [−1.81, 0.51] | 0.269 |  |  |  |
| PD-RT | 1 | Age | −0.24 | −0.20 | [−0.51, 0.02] | 0.067 | 0.039 | — | 3.44 |
|  | 2 | Age | −0.24 | −0.20 | [−0.51, 0.02] | 0.073 | 0.040 | 0.000 | 1.71 |
|  |  | 2-Min | 0.01 | 0.02 | [−0.09, 0.10] | 0.875 |  |  |  |
| Diffusion model parameters | | | | | | | | | |
| Drift rate (*v*) | 1 | Age | 0.006 | 0.15 | [−0.003, 0.015] | 0.17 | 0.022 | — | 1.92 |
|  | 2 | Age | 0.007 | 0.18 | [−0.001, 0.016] | 0.086 | 0.09 | 0.068 | 6.35* |
|  |  | 2-Min | 0.004 | 0.26 | [0.001, 0.007] | 0.014 |  |  |  |
| Boundary separation (*a*) | 1 | Age | 0.007 | 0.28 | [0.002, 0.012] | 0.009 | 0.077 | — | 7.19** |
|  | 2 | Age | 0.007 | 0.26 | [0.001, 0.012] | 0.014 | 0.091 | 0.013 | 1.25 |
|  |  | 2-Min | −0.001 | −.12 | [−0.003, 0.001] | 0.266 |  |  |  |
| Non-decision time (*T_er_*) | 1 | Age | 0.001 | 0.12 | [−0.001, 0.003] | 0.274 | 0.014 | — | 1.21 |
|  | 2 | Age | 0.001 | 0.13 | [−0.001, 0.003] | 0.24 | 0.02 | 0.006 | 0.55 |
|  |  | 2-Min | 0 | 0.08 | [−0.000, 0.001] | 0.46 |  |  |  |

Note. *M* = mean; *SD* = standard deviation; 2-Min = 2-minute Step Test; Mean-RT = mean reaction time; CV-RT = coefficient of variation of reaction time; Slow-RT = slowest 10% reaction time; PD-RT = performance decrement of reaction time. ^*^ *p* < .05; ^**^ *p* < .01; ^***^ *p* < .001
